# Mortality trends for diabetes mellitus, hypertension and cardiovascular disease among people living with and without HIV in Brazil during the COVID‐19 pandemic, 2020–2022

**DOI:** 10.1111/hiv.70240

**Published:** 2026-04-08

**Authors:** Tatyellen Natasha da Costa Oliveira, Rodrigo C. Moreira, Luiz Max Carvalho, Flávio Codeço Coelho, Marcelo Ferreira da Costa Gomes, Leonardo S. Bastos, Laís Picinini Freitas, Antonio G. Pacheco

**Affiliations:** ^1^ Escola Nacional de Saúde Pública Sergio Arouca, Fundação Oswaldo Cruz Rio de Janeiro Brazil; ^2^ Seção de Ensino e Informação Científica Instituto Evandro Chagas Ananindeua Brazil; ^3^ Instituto Nacional de Infectologia Evandro Chagas Fundação Oswaldo Cruz Rio de Janeiro Brazil; ^4^ Fundação Getúlio Vargas Rio de Janeiro Brazil; ^5^ Programa de Computação Científica Fundação Oswaldo Cruz Rio de Janeiro Brazil; ^6^ Departamento de Epidemiologia e Métodos Quantitativos em Saúde Vice Direção de Pesquisa e Inovação Escola Nacional de Saúde Pública Sergio Arouca, Fundação Oswaldo Cruz Rio de Janeiro Brazil

**Keywords:** cardiovascular diseases, COVID‐19, diabetes mellitus, HIV, hypertension, mortality

## Abstract

**Introduction:**

People living with HIV (PLWH) face an increasing burden of non‐AIDS‐related chronic conditions. However, how the COVID‐19 pandemic affected chronic disease‐related mortality in this population remains unclear. We assessed changes in mortality among PLWH in Brazil during the COVID‐19 pandemic.

**Methods:**

We conducted a nationwide analysis using Brazil's Mortality Information System, including adults aged ≥18 years with and without HIV mentioned on death certificates from 2016 to 2022. Age‐ and sex‐adjusted mortality ratios (aMRs) were estimated for all‐cause mortality and for deaths associated with diabetes mellitus (DM), hypertension (HTN) and cardiovascular disease (CVD) using Poisson generalized linear mixed models, with the pre‐pandemic period 2016–2019 used as the reference.

**Results:**

Between 2016 and 2022, 90 888 deaths occurred among PLWH and 9 723 490 among individuals without HIV. Among PLWH, adjusted all‐cause mortality ratios were lower in 2020 (aMR 0.918; 95% CI 0.900–0.936) and 2022 (aMR 0.950; 95% CI 0.932–0.968), while estimates for 2021 were close to the reference period. In contrast, individuals without HIV experienced increased mortality in 2020 (aMR 1.106; 95% CI 1.104–1.108), 2021 (aMR 1.269; 95% CI 1.267–1.272) and 2022 (aMR 1.029; 95% CI 1.027–1.031). Among PLWH, mortality associated with DM (aMRs 1.573–1.666), HTN (aMRs 1.738–1.978) and CVD (aMRs 1.242–1.377) increased consistently across all pandemic years. Among individuals without HIV, chronic disease‐related mortality showed greater temporal variability, with increases in 2020 and 2021 followed by reductions in 2022.

**Conclusions:**

Chronic disease‐related mortality increased persistently among PLWH during the COVID‐19 pandemic, contrasting with declining trends in the general population. These results emphasize distinct mortality dynamics among PLWH during public health crises.

## INTRODUCTION

In 2020, the Coronavirus Disease (COVID‐19) pandemic, caused by severe acute respiratory syndrome coronavirus 2 (SARS‐CoV‐2), emerged as a global health crisis. During 2020 and 2021, excess mortality reached alarming levels worldwide [1], with Brazil reporting significant regional disparities in mortality burden [2, 3].

Beyond the direct impact of COVID‐19, the pandemic disrupted health systems and intensified existing social and structural vulnerabilities, particularly among individuals with preexisting health conditions. These groups were disproportionately affected by overwhelmed healthcare services, interruptions in routine care, reduced access to essential treatments and diagnostics and fear of seeking medical attention [4, 5]. Chronic conditions such as diabetes mellitus (DM), hypertension (HTN) and cardiovascular diseases (CVD) are highly prevalent and were strongly associated with increased mortality during the pandemic period [6, 7, 8].

In this context, people living with HIV (PLWH) represent a population of interest. With the success of antiretroviral therapy (ART), life expectancy has increased [9, 10], but non‐AIDS‐related chronic comorbidities, such as CVD and DM, have become more prevalent, requiring continuous engagement with healthcare services [10, 11, 12, 13, 14, 15, 16, 17, 18, 19]. This need for continuous interaction with the healthcare system to manage multiple morbidities may have increased PLWH's vulnerability to both the direct and indirect consequences of the COVID‐19 pandemic, raising concerns about excess mortality in this group [20, 21].

Although findings have varied across studies, population‐based evidence suggests that PLWH, particularly those with advanced immunosuppression or low CD4^+^ T‐cell counts, may have experienced a higher risk of severe COVID‐19 and COVID‐19‐related mortality compared with individuals without HIV [22, 23].

In Brazil, an estimated 1 122 723 people were living with HIV in 2022 [24], of whom 84.2% were linked to HIV care and 76.4% were retained in care. Overall ART coverage reached 73.1%, and viral suppression (HIV RNA <50 copies/mL) was achieved in 65.4% of all PLWH [25]. Despite these advances at the population level, substantial social and regional inequalities in access to care persist. Excess mortality in Brazil during the COVID‐19 pandemic has been examined in the general Brazilian population [2, 7], but to our knowledge, no prior population‐based study in Brazil has assessed excess mortality among PLWH across multiple pandemic years, either overall or in relation to chronic noncommunicable conditions.

We hypothesize that PLWH experienced a disproportionate increase in mortality during the COVID‐19 pandemic, particularly associated with chronic conditions such as CVD, DM and HTN. To investigate this, we compared annual adjusted mortality ratios (aMRs) from 2020 to 2022 with the pre‐pandemic period (2016–2019) among individuals with and without mention of HIV on their death certificates. Additional analyses explored whether similar patterns were observed for deaths associated with CVD, DM and HTN.

## METHODS

Mortality data were extracted from the Brazilian mortality system (SIM–Sistema de Informação sobre Mortalidade), a nationwide system that compiles death certificate data for the entire country. In Brazil, death registration is legally mandatory, and the SIM has coverage estimates approaching 98% in evaluations comparing SIM data to other administrative sources [26], supporting its use for population‐based mortality analyses. The database is publicly available through DATASUS (Opendatasus—https://opendatasus.saude.gov.br/dataset/sim).

Complete mortality files from January 2016 to December 2022, including information on underlying and multiple causes of death listed in the death certificates (DC), were downloaded (accessed on 03/27/2024) and processed as described below. Causes of death were coded according to the International Classification of Diseases, 10th Revision (ICD‐10).

Population data were obtained from the Brazilian Institute of Geography and Statistics (IBGE – Instituto Brasileiro de Geografia e Estatística) through the SIDRA system (http://api.sidra.ibge.gov.br/). Annual population projections stratified by age group, sex and federative unit of residence from January 2016 to December 2022 were used.

### Multiple cause of death approach and outcome definitions

In this study, we adopted a multiple cause of death (MCoD) approach [27, 28], which considers information from the underlying, immediate, contributing and concomitant causes leading to death, to better capture the burden of chronic conditions that often contribute to the fatal event but are not selected as the underlying cause of death. This approach has been previously used in studies among PLWH worldwide [29, 30, 31] and in Brazil [10, 11, 12, 19].

Therefore, any mention of the following ICD‐10 codes in the fields where causes of death are recorded (i.e., Part VI‐49 of the DC) was used to identify PLWH and deaths associated with the condition of interest:

- HIV—ICDs: B20 to B24 and Z21
- DM—ICDs: E10 to E14
- HTN—ICDs: I10 to I15
- CVD—ICDs: I00 to I99, except I46 (cardiac arrest)

ICD‐10 codes specific to COVID‐19 were not included as outcomes, as the analytical focus was on mortality associated with chronic noncommunicable conditions during the pandemic period.

Because the MCoD approach allows multiple conditions to be recorded on the same death certificate, a single individual could contribute to more than one outcome category (e.g., DM‐ and CVD‐associated mortality). Therefore, the aMRs estimated in this study should be interpreted as condition‐associated mortality, rather than mutually exclusive, cause‐specific mortality based solely on the underlying cause of death.

Death certificates of individuals under 18 years of age were excluded from the analysis due to the low prevalence of the condition of interest in this study within that age group.

### Statistical analysis

aMRs were estimated for each calendar year during the pandemic period (2020, 2021 and 2022) using the pre‐pandemic period of 2016–2019 as the reference. In this study, excess mortality was operationalized as a relative measure, defined as aMR values greater than one compared with the reference period. This approach captures changes in mortality risk during the pandemic relative to a recent pre‐pandemic baseline and has been employed in a previous study assessing excess mortality associated with chronic conditions during the COVID‐19 period in Brazil [7].

A generalized linear mixed modeling approach with a Poisson distribution and a log link function was adopted to estimate the aMRs. This model was selected due to the hierarchical nature of the data, in which deaths are nested within federative units and influenced by contextual factors at the federative unit level. The natural logarithm of the population size by year, age group, sexand federative unit was included as an offset, allowing the model to estimate rates instead of counts.

All models included calendar year (categorical: 2016–2019 [reference], 2020, 2021, 2022), age group (categorical: 18–29 [reference], 30–39, 40–49, 50–59, 60–69, 70–79, 80+) and sex (categorical: female [reference], male) as fixed effects and federative unit of residence as a random intercept to account for unobserved heterogeneity and regional differences.

Separate models were fitted for each outcome (overall mortality, DM‐, HTN‐ and CVD‐associated mortality) and for each group (PLWH and general population). Full model specifications are available at Data S1.

Model assumptions, including equidispersion, normality of random effects and convergence, were evaluated.

All analyses were performed using the R language (version 4.3.2) and the RStudio integrated development environment (version 2023.12.0) [32, 33].

### Ethics Statement

This research utilized publicly available information and aggregated data without individual identification (DataSUS and IBGE data) and was approved by the Research Ethics Committee of the Sergio Arouca National School of Public Health, Oswaldo Cruz Foundation (ENSP/Fiocruz; number 7.210.374).

## RESULTS

A total of 9 814 378 deaths among adults (≥18 years) were recorded in Brazil between 2016 and 2022. Overall, 90 888 deaths occurred among PLWH, and 9 723 490 among individuals without HIV.

A descriptive overview of all recorded adult deaths, stratified by HIV status, age group, sex, year of death and associated causes, including DM, CVD, HTN and COVID‐19, is presented in Table 1. Mortality was consistently higher among males in both groups, accounting for 55.34% of deaths among individuals without HIV and 66.65% among PLWH. Age distribution differed markedly between groups. Among individuals without HIV, the largest proportion of deaths occurred among those aged 80 years and older (31.08%), whereas among PLWH, most deaths occurred between 30 and 59 years of age.

**TABLE 1:** Characteristics of mortality data in Brazil stratified by mention of HIV in their death certificate, 2016–2022.

Variables	HIV	No HIV	*p* value^a^
	*N* = 90 888	*N* = 9 723 490	
Sex			<0.001
Male	60 563 (66.65%)	5 380 192 (55.34%)	
Female	30 307 (33.35%)	4 342 534 (44.66%)	
Age group			<0.001
18–29	10 347 (11.38%)	429 410 (4.42%)	
30–39	21 589 (23.75%)	445 246 (4.58%)	
40–49	24 999 (27.51%)	686 045 (7.06%)	
50–59	19 231 (21.16%)	1 194 078 (12.28%)	
60–69	10 081 (11.09%)	1 806 388 (18.58%)	
70–79	3630 (3.99%)	2 140 589 (22.01%)	
80+	1011 (1.11%)	3 021 734 (31.08%)	
Diabetes mellitus			<0.001
No	87 862 (96.67%)	8 535 786 (87.79%)	
Yes	3026 (3.33%)	1 187 704 (12.21%)	
Cardiovascular disease			<0.001
No	79 714 (87.71%)	5 418 748 (55.73%)	
Yes	11 174 (12.29%)	4 304 742 (44.27%)	
Hypertension			<0.001
No	87 000 (95.72%)	7 549 933 (77.65%)	
Yes	3888 (4.28%)	2 173 557 (22.35%)	
COVID‐19			<0.001
No	86 765 (95.46%)	8 985 418 (92.41%)	
Yes	4123 (4.54%)	738 072 (7.59%)	
Year			<0.001
2016–2019	51 877 (57.08%)	4 989 164 (51.31%)	
2020	12 371 (13.61%)	1 491 995 (15.34%)	
2021	13 469 (14.82%)	1 766 231 (18.16%)	
2022	13 171 (14.49%)	1 476 100 (15.18%)	

^a^ Pearson's chi‐squared test.

Additional stratification by state of residence is provided in Table S1.

**FIGURE 1:**
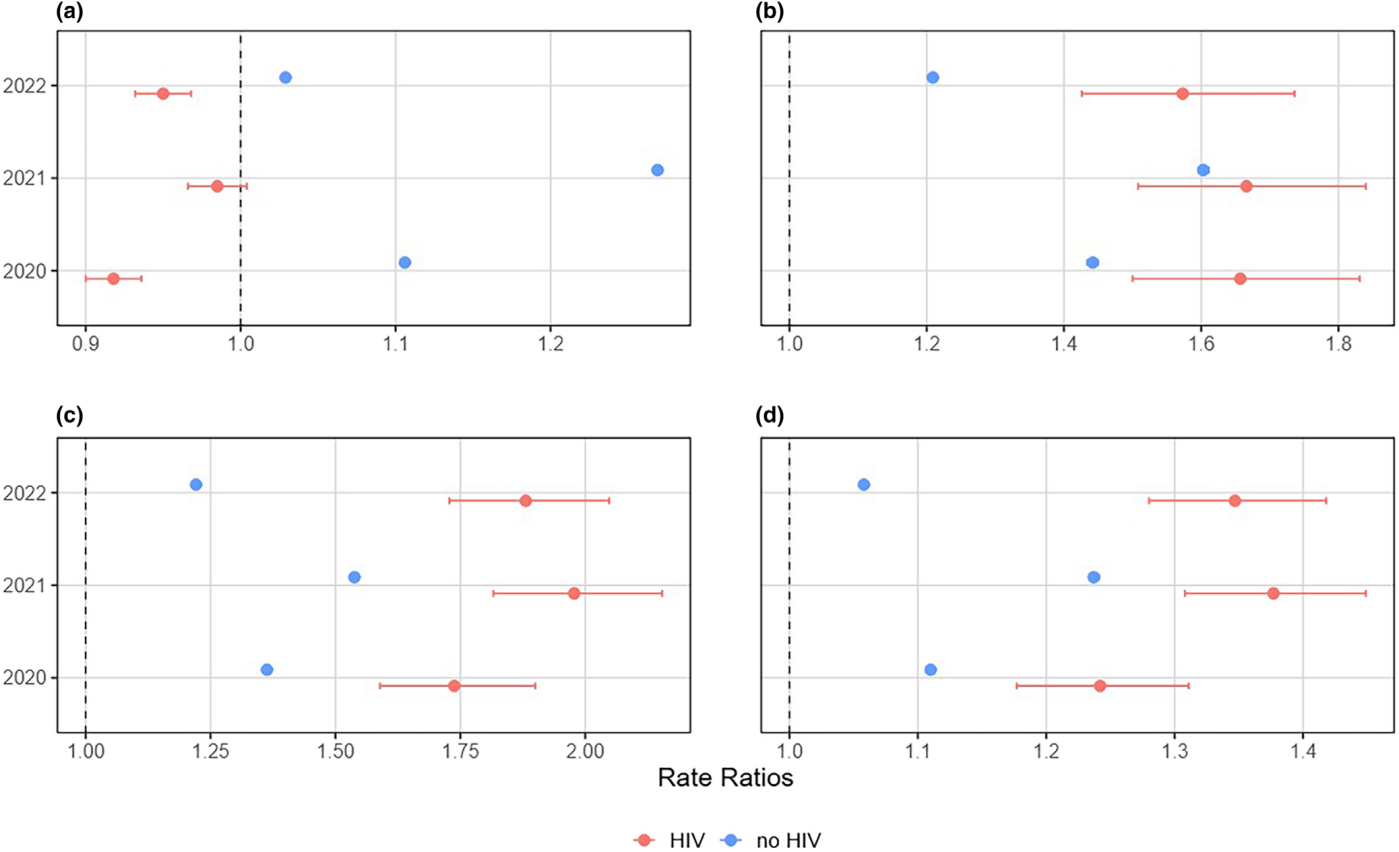
Adjusted Mortality Ratios among people with and without HIV (a) overall, and with a mention of (b) diabetes mellitus, (c) hypertension, (d) cardiovascular disease on the death certificate, 2020–2022 compared with 2016–2019, Brazil.

In general, aMRs among individuals without HIV were higher in 2020 (10%), 2021 (27%) and 2022 (2%) compared with the 2016–2019 reference period (Table 2 and Figure 1a). Among individuals with HIV mentioned on the DC, no statistically significant increase in adjusted all‐cause mortality was observed in 2021. However, adjusted mortality decreased by 8% in 2020 and by 5% in 2022, indicating lower mortality relative to the pre‐pandemic period. Across all years, aMRs among PLWH were consistently lower than those observed among individuals without HIV (Table 2).

**TABLE 2:** Adjusted mortality ratio comparisons among individuals who had diabetes mellitus (DM), hypertension (HTN) or cardiovascular disease (CVD) mentioned in their death certificate with and without mention of HIV, from 2020 to 2022 in Brazil.

Group	Category	2020		2021		2022	
		aMR	CI 95%	aMR	CI 95%	aMR	CI 95%
Total	HIV	0.918	0.900–0.936	0.985	0.966–1.004	0.950	0.932–0.968
	No HIV	1.106	1.104–1.108	1.269	1.267–1.272	1.029	1.027–1.031
DM	HIV	1.657	1.500–1.831	1.666	1.508–1.840	1.573	1.426–1.736
	No HIV	1.442	1.434–1.449	1.603	1.596–1.611	1.209	1.203–1.215
HTN	HIV	1.738	1.589–1.900	1.978	1.816–2.154	1.881	1.728–2.048
	No HIV	1.363	1.358–1.368	1.538	1.532–1.543	1.221	1.216–1.225
CVD	HIV	1.242	1.177–1.311	1.377	1.308–1.449	1.347	1.280–1.418
	No HIV	1.110	1.107–1.113	1.237	1.234–1.241	1.058	1.055–1.061

*Note*: aMR Adjusted by sex, age group as fixed effect and state of residence as random effect. All models converged successfully without optimization errors, the random effects followed a normal distribution, and their structure was identifiable and contributed meaningfully to model fit.

Among individuals without HIV, aMRs associated with DM increased by 44.2% in 2020, 60.3% in 2021 and 20.9% in 2022 compared to 2016–2019. (Figure 1a). This indicates a substantial increase in mortality among the diabetic population without HIV, particularly noted in 2021, which saw the most pronounced rise.

Among individuals with HIV and DM mentioned on their DC, the increase in adjusted mortality rates was 65.7%, 66.6% and 57.3% for 2020, 2021 and 2022, respectively. These increases were statistically significant and showed a relatively stable pattern across the 3 years, in contrast to the year‐to‐year variability observed among individuals without HIV (Figure 1b).

HTN‐associated mortality increased in both groups throughout the pandemic period. Among PLWH, adjusted mortality rates increased between 73.8% and 97.8%, while among individuals without HIV, they increased between 22.1% and 53.8%. Although HTN‐associated mortality was higher than the pre‐pandemic baseline in all years for both groups, aMRs were consistently and statistically significantly higher among PLWH compared with individuals without HIV throughout the pandemic period. This pattern was observed in 2020, 2021 and 2022, indicating a persistently greater increase in HTN‐associated mortality among PLWH relative to individuals without HIV (Figure 1c).

For deaths with mention of CVD on their DC, aMRs increased in both groups across all pandemic years and followed the same pattern of statistical significance observed for HTN, with consistently higher estimates among PLWH compared with the general population. In 2020, the adjusted CVD‐associated mortality rate was 24.2% higher among individuals with HIV and 11.0% among those without HIV. In 2021, increases remained consistent between the groups, with an excess of 37.7% for individuals with HIV and 23.7% for those without HIV. In 2022, however, adjusted CVD‐associated mortality remained elevated among PLWH (34.7%), while a smaller increase was observed among individuals without HIV (5.8%) (Figure 1d).

**FIGURE 2:**
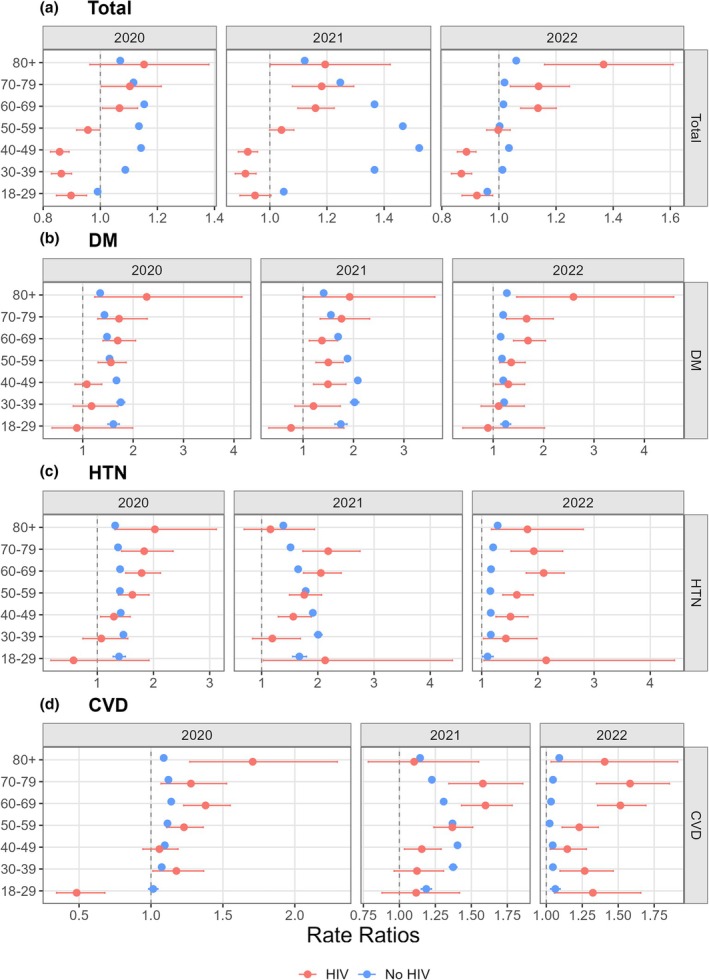
Adjusted Mortality Ratios stratified by age among people with and without HIV (a) overall, and with a mention of (b) diabetes mellitus, (c) hypertension, (d) cardiovascular disease on the death certificate, 2020–2022 compared with 2016–2019, Brazil.

Overall age‐stratified analysis of aMRs reveals that older demographic segments of PLWH appeared to align with mortality ratios observed in the population without HIV in 2020 and 2021 (Figure 2a) but not younger individuals. During the same period, aMRs for age groups between 18 and 69 years were higher among people without HIV when compared to PLWH. Furthermore, in 2022, a distinct divergence was observed in some middle‐aged groups, particularly between 30 and 49 years, where aMRs were higher among individuals without HIV compared with PLWH, as indicated by non‐overlapping 95% confidence intervals.

For DM and HTN, only specific differences were statistically significant in the age‐group stratified analysis. Specifically, PLWH aged 30 to 49 in the year 2021 had a lower DM‐associated aMR compared to the same age group without HIV (Figure 2b); a similar scenario was observed in the age group of 30 to 39 for HTN‐associated aMR in the year 2021 (Figure 2c). In 2022, a difference was observed for HTN‐associated mortality among individuals aged 50–79 years, where aMRs among PLWH were higher than those observed in individuals without HIV.

aMRs associated with CVD were similar between people with and without HIV, except for the age groups 60 to 69 and 80+ in 2020, 40 to 49 in 2021 and 60 to 79 in 2022, where those without HIV showed lower aMRs compared to PLWH in the same age group. Additionally, among those aged 40–49 years in 2021, PLWH exhibited lower aMR than those without HIV in the same age groups (Figure 2d).

## DISCUSSION

The findings of this study revealed an intriguing dynamic in aMRs among individuals with and without mention of HIV on their DC, both overall and associated with conditions such as DM, HTN and CVD. Among individuals with HIV mentioned on the death certificate, no statistically significant increase in overall mortality was observed during the pandemic years. Instead, aMRs were significantly lower in 2020 (8% lower) and 2022 (5% lower) compared with the 2016–2019 reference period, while estimates in 2021 were not statistically different from the pre‐pandemic baseline. On the other hand, for the group without HIV, aMRs showed a significant increase in mortality rates in 2020 and 2021, followed by a reduction in 2022, although still above the rate of the reference period. These observations are noteworthy. Although PLWH have a higher prevalence of chronic comorbidities [10, 11, 12, 13, 14, 15, 16, 17, 18, 19], this does not necessarily translate into higher overall mortality, possibly reflecting earlier diagnosis and continuous clinical monitoring in this population.

A large study conducted in the United States identified similar results: mortality rates in the general population increased significantly in 2020 compared to 2019 and 2018, with an increase of over 18.1%, while among PLWH, a 9.3% increase was observed, though it was not statistically significant [34].

Other findings in the literature have suggested that, after controlling for underlying differences in mortality between PLWH and the general population, living with HIV was not associated with a statistically significant increase in overall mortality, and no clinically meaningful differences were observed [6, 35, 36].

The substantial impact of the COVID‐19 pandemic on the overall mortality rate of individuals without HIV was confirmed in previous studies that pointed to an increase in mortality rates during periods of healthcare system overload during the COVID‐19 pandemic [2, 3, 7, 37].

However, the lack of an increase in mortality among PLWH during the pandemic years, especially considering the higher baseline prevalence of comorbidities in this population, is noteworthy and warrants further examination. One possible explanation is the underreporting or misclassification of HIV status or related causes of death during the early stages of the pandemic, when the health system was under intense pressure and diagnostic priorities were redirected toward COVID‐19 [38]. This may have led to an underestimation of HIV‐associated mortality in 2020 and should be considered when interpreting these findings.

Moreover, given the chronic nature of HIV and the need for continuous management of the condition, it is plausible that these individuals developed a heightened awareness of the importance of non‐pharmaceutical interventions, which may have translated into more stringent adherence to COVID‐19 prevention measures, as well as an effort to maintain follow‐up and treatment of their chronic conditions, as suggested in previous studies [39, 40].

Our findings revealed a substantial increase in aMRs for individuals with DM mentioned on their DC, both among PLWH and those without HIV, during the COVID‐19 pandemic period. While mortality for individuals with DM and HIV remained high and relatively constant over the years analyzed, approximately 60% higher than the 2016–2019 period, the non‐HIV population experienced more pronounced fluctuations in DM‐associated mortality. A previous study demonstrated that the majority of the excess mortality due to DM among the Brazilian population in the years 2020, 2021 and 2022 is related to COVID‐19 [7].

Therefore, a possible explanation for the decline in DM‐associated mortality observed in 2022 may be the initiation of Brazil's COVID‐19 vaccination campaign in January 2021 and the achievement of broad two‐dose coverage by December of that year [41]. The beneficial effects of COVID‐19 vaccination in reducing mortality among individuals with type 1 and type 2 diabetes have been documented in previous studies [42].

This effect, however, was not observed among individuals with mention of both HIV and DM on their DC, with excess mortality in this group remaining consistently about 57%–67% higher than before the pandemic throughout the entire study period. One possible explanation for this persistent elevation may be related to differential vaccine response in PLWH. Although PLWH may achieve similar immune responses to HIV‐negative individuals after booster doses, initial seroconversion rates, particularly among those with lower CD4+ counts or higher viral loads, tend to be lower [43, 44]. Moreover, studies have shown that fully vaccinated PLWH still faced a higher risk of COVID‐19 diagnosis and death compared to HIV‐negative individuals [45, 46].

The increase in mortality rates associated with DM and other non‐AIDS‐related causes among PLWH had already been documented in previous studies conducted before the COVID‐19 pandemic, indicating a growing burden of non‐immunodeficiency‐related conditions in this population [10, 19]. However, during the pandemic period, the magnitude of this increase was substantially greater, suggesting that the health crisis may have exacerbated pre‐existing trends and further intensified vulnerabilities among PLWH.

The increase in mortality rates associated with HTN and CVD showed a similar pattern. Although mortality associated with HTN and CVD increased in both groups relative to the pre‐pandemic period, PLWH consistently presented statistically significantly higher aMRs than those without HIV. Similar to the pattern previously observed for DM, previous studies have demonstrated increasing trends of mortality rates for CVD and HTN among PLWH [10, 12, 19].

The increases and sustained elevation in mortality associated with DM, HTN and CVD among PLWH during the pandemic highlight a growing vulnerability that extends beyond HIV‐related risks. These results highlight the need to move beyond vertical, disease‐specific approaches and toward integrated care models that combine HIV management with proactive screening, prevention and treatment of chronic non‐communicable diseases.

Since 2016, the World Health Organization has recommended that cardiovascular risk assessment be incorporated into routine HIV care, particularly in low‐ and middle‐income countries [47]. In Brazil, despite the broad availability of ART through the SUS, structural inequalities and regional disparities continue to limit access to comprehensive chronic disease management. Although Brazil has a well‐established Clinical Protocol and Therapeutic Guidelines for the management of HIV infection [48], there is no specific national protocol for the routine monitoring of chronic conditions such as DM, HTN or CVD in this population. Current recommendations are limited to individualized clinical follow‐up and attention to the development of insulin resistance or cardiovascular disease during the course of HIV treatment [48].

Strengthening the integration between HIV services and primary care, ensuring continuity of treatment during health system disruptions and expanding routine risk assessments for cardiovascular and metabolic conditions are essential steps to improve outcomes in this population.

Globally, as the PLWH population ages, health systems must be prepared to address the dual burden of HIV and chronic comorbidities. The COVID‐19 pandemic has exposed the fragility of fragmented care models and reinforced the importance of health system resilience, equitable resource allocation and person‐centered strategies that prioritize continuity of care for vulnerable populations [39, 40, 49, 50, 51].

This study has several limitations. First, the unavailability of consistent data in the Mortality Information System limited the inclusion of relevant variables such as race/ethnicity, education and clinical characteristics. However, adjustments for sex, age group and state of residence were considered sufficient to account for major sources of heterogeneity in our data. Further research is needed to explore associations with additional variables.

Second, there is the possibility of misclassification of HIV status on DC due to underreporting, as well as inaccuracies in recording the underlying or contributing causes of death. These issues could result in an underestimation of the conditions investigated in this study (DM, CVD, HTN). To mitigate this, we adopted a multiple‐cause‐of‐death approach, searching for relevant ICD‐10 codes across all fields of the death certificate. This method has been widely applied in studies of cause‐of‐death patterns both globally [29, 30, 31] and in Brazil [10, 11, 12, 19]. However, relying on death certificate data inherently introduces risks of misclassification and underreporting. The quality of cause‐of‐death attribution may vary by region, depending on the certifier's training, local reporting practices and health system infrastructure. We believe that if these mistakes did happen, they likely affected both groups in a similar way, potentially leading to conservative estimates of condition‐associated mortality. In addition, social stigma related to HIV may contribute to underreporting of HIV status on death certificates in some cases, potentially leading to further underestimation of HIV‐associated mortality.

Third, excess mortality can be estimated using different methodological approaches, including comparisons with multi‐year baseline averages [38, 52] or model‐based expected mortality estimates [2, 53, 54]. In this study, we defined excess mortality as mortality rates above those observed in the pre‐pandemic reference period (2016–2019), consistent with previous studies using similar approaches [3, 7]. However, alternative definitions may yield different magnitude estimates and should be considered when comparing findings across studies.

Lastly, although the study covers eight consecutive years (2016–2022), it did not capture the long‐term effects of the COVID‐19 pandemic on mortality among PLWH. Continued monitoring is needed to assess whether the observed trends persist, worsen or stabilize in the post‐pandemic period. Future studies should also incorporate clinical data, such as treatment regimens, disease control markers and socioeconomic variables, to enhance the interpretation of mortality differentials.

Despite these limitations, this study presents important strengths. It utilizes a comprehensive, nationwide dataset from Brazil's mortality information system, enabling a population‐level assessment of mortality trends across multiple years. The use of a multiple cause of death strategy allowed for a more complete capture of mortality associated with chronic conditions in both PLWH and individuals without HIV, offering a detailed representation of the evolving burden of disease during the COVID‐19 pandemic.

## CONCLUSIONS

This study revealed a significant rise in aMRs for chronic conditions such as DM, HTN and CVD, both among PLWH and those without HIV during the COVID‐19 pandemic. Although overall mortality among PLWH did not increase to the same extent as observed in the general population, mortality associated with chronic non‐communicable diseases increased and remained consistently elevated compared to the pre‐pandemic period, pointing out a shifting burden of disease that extends beyond HIV itself.

These findings emphasize the need for public health systems to adopt more integrated and resilient care models, capable of addressing the dual burden of infectious and chronic diseases, particularly during periods of health system disruption. Strengthening the integration between HIV services and primary care, expanding cardiovascular risk screening and ensuring equitable access to essential treatments should be prioritized in health policy agendas focused on PLWH.

## AUTHOR CONTRIBUTIONS

TNCO, RCM: Responsible for literature search, data analysis and interpretation, drafting the initial manuscript and revising, including pre‐ and post‐publication stages. LMC, FCC, MFCG, LSB, LPF: Contributed to writing, reviewing and editing, including the preparation of the original manuscript, critical analysis, comments and revisions during the pre‐ and post‐publication stages. AGP: Senior author, was responsible for supervision and leadership in the planning and execution of the research, overseeing all stages of the process, providing strategic guidance throughout the research process and reviewing and approving the final version of the manuscript. Additionally, the senior author contributed to the application of statistical techniques to analyze and synthesize the study data.

## CONFLICT OF INTEREST STATEMENT

The authors declare that they have no competing interests related to this study.

## Supporting information


**Data S1.** General model specification used to estimate aMRs.

**Table S1.** Characteristics of mortality data in Brazil, 2016–2022.

## Data Availability

The data that support the findings of this study are openly available in Opendatasus at https://opendatasus.saude.gov.br/dataset/sim and the Brazilian Institute of Geography and Statistics (IBGE–Instituto Brasileiro de Geografia e Estatística) through the SIDRA system at http://api.sidra.ibge.gov.br/.
