# Supplementary material for: Mortality trends for diabetes mellitus, hypertension and cardiovascular disease among people living with and without HIV in Brazil during the COVID‐19 pandemic, 2020–2022
Source: HIV Med. 2026 Apr 8;27(8):1265–76. doi: 10.1111/hiv.70240 (PMC13432445; doi:10.1111/hiv.70240)
Supplement: Supplementary file 1 — Data S1. General model specification used to estimate aMRs. [file HIV-27-1265-s001.docx]

**Document S1 - General model specification used to estimate aMRs**

$$\ln\left( \mu_{ij} \right)=\beta_{0j}+\sum_{k=1}^{3} \beta_{1,k}{period}_{k,ij}+\beta_{2}{sex}_{ij}+\sum_{k=1}^{6} \beta_{3,k}{agegroup}_{k,ij}+\ln\left( {Pop}_{ij} \right)+\epsilon_{ij}$$

$$\beta_{0j}=\beta_{0}+u_{j}$$

$$u_{0j} \sim N \left( {0,\sigma}^{2} \right)$$

𝜇_ij_ is the expected number of deaths in stratum *i* (defined by the combination of year, age group, and sex) within federative unit *j*;

β_0_ is the fixed intercept, representing the baseline mortality rate for the reference category (period 2016-2019, females aged 18–29);

β_1,k_ are the fixed-effect coefficient for year, where k=1,2,3 correspond to the years 2020, 2021 and 2022, respectively (reference category: 2016-2019);

β_2_ is the fixed-effect coefficient for sex;

β_3,k_ are the fixed-effect coefficients for age group, where k=1,2,3,4,5,6 correspond to the age groups 30-39, 40-49, 50-59, 60-69, 70-79 and 80+, respectively (reference category: 18−29);

Pop_ij_ is the population size in the same stratum, included as an offset on the logarithmic scale;

u_j_ is the random intercept for federative unit j, assumed to follow a normal distribution with mean 0 and variance σ^2^.

**Additional Notes**

The model uses log link (standard in Poisson models).

Inclusion of population as a log-offset allows modeling rates instead of counts.

The random intercept (u_j_) allows for unobserved heterogeneity across federative units.
