# Supplementary material for: Mortality trends for diabetes mellitus, hypertension and cardiovascular disease among people living with and without HIV in Brazil during the COVID‐19 pandemic, 2020–2022
Source: HIV Med. 2026 Apr 8;27(8):1265–76. doi: 10.1111/hiv.70240 (PMC13432445; doi:10.1111/hiv.70240)
Supplement: Supplementary file 2 — Table S1. Characteristics of mortality data in Brazil, 2016–2022. [file HIV-27-1265-s002.docx]

**Table S1 - Characteristics of mortality data in Brazil, 2016-2022.**

| **Variable** | **Year** | | | |
| --- | --- | --- | --- | --- |
|  | **2016-2019**,  N = 5,041,041 | **2020**,  N = 1,504,366 | **2021**,  N = 1,779,700 | **2022**,  N = 1,489,271 |
| **Sex** |  |  |  |  |
| Male | 2,800,794 (55.56%) | 842,955 (56.04%) | 984,118 (55.30%) | 812,888 (54.59%) |
| Female | 2,239,846 (44.44%) | 661,314 (43.96%) | 795,419 (44.70%) | 676,262 (45.41%) |
| **Age group** |  |  |  |  |
| 18-29 | 253,090 (5.02%) | 62,011 (4.12%) | 65,290 (3.67%) | 59,366 (3.99%) |
| 30-39 | 249,775 (4.95%) | 68,164 (4.53%) | 85,202 (4.79%) | 63,694 (4.28%) |
| 40-49 | 359,610 (7.13%) | 106,488 (7.08%) | 143,975 (8.09%) | 100,971 (6.78%) |
| 50-59 | 620,925 (12.32%) | 184,102 (12.24%) | 240,690 (13.52%) | 167,592 (11.25%) |
| 60-69 | 911,014 (18.07%) | 286,190 (19.02%) | 350,131 (19.67%) | 269,134 (18.07%) |
| 70-79 | 1,086,191 (21.55%) | 334,733 (22.25%) | 390,064 (21.92%) | 333,231 (22.38%) |
| 80+ | 1,560,436 (30.95%) | 462,678 (30.76%) | 504,348 (28.34%) | 495,283 (33.26%) |
| **DM** |  |  |  |  |
| No | 4,499,586 (89.26%) | 1,291,465 (85.85%) | 1,534,648 (86.23%) | 1,297,949 (87.15%) |
| Yes | 541,455 (10.74%) | 212,901 (14.15%) | 245,052 (13.77%) | 191,322 (12.85%) |
| **CVD** |  |  |  |  |
| No | 2,840,245 (56.34%) | 838,191 (55.72%) | 1,011,020 (56.81%) | 809,006 (54.32%) |
| Yes | 2,200,796 (43.66%) | 666,175 (44.28%) | 768,680 (43.19%) | 680,265 (45.68%) |
| **HTN** |  |  |  |  |
| No | 4,035,547 (80.05%) | 1,129,894 (75.11%) | 1,342,022 (75.41%) | 1,129,470 (75.84%) |
| Yes | 1,005,494 (19.95%) | 374,472 (24.89%) | 437,678 (24.59%) | 359,801 (24.16%) |
| **HIV** |  |  |  |  |
| No | 4,989,164 (98.97%) | 1,491,995 (99.18%) | 1,766,231 (99.24%) | 1,476,100 (99.12%) |
| Yes | 51,877 (1.03%) | 12,371 (0.82%) | 13,469 (0.76%) | 13,171 (0.88%) |
| **State of residence** |  |  |  |  |
| AC | 14,010 (0.28%) | 4,425 (0.29%) | 5,046 (0.28%) | 3,766 (0.25%) |
| AL | 76,199 (1.51%) | 23,157 (1.54%) | 24,044 (1.35%) | 22,099 (1.48%) |
| AM | 61,810 (1.23%) | 22,979 (1.53%) | 27,130 (1.52%) | 18,226 (1.22%) |
| AP | 11,156 (0.22%) | 4,223 (0.28%) | 4,280 (0.24%) | 3,469 (0.23%) |
| BA | 341,110 (6.77%) | 102,623 (6.82%) | 110,760 (6.22%) | 102,987 (6.92%) |
| CE | 215,432 (4.27%) | 66,992 (4.45%) | 71,372 (4.01%) | 62,350 (4.19%) |
| DF | 46,655 (0.93%) | 15,666 (1.04%) | 18,493 (1.04%) | 13,854 (0.93%) |
| ES | 90,507 (1.80%) | 28,087 (1.87%) | 31,682 (1.78%) | 26,930 (1.81%) |
| GO | 149,976 (2.98%) | 46,557 (3.09%) | 58,885 (3.31%) | 45,373 (3.05%) |
| MA | 128,007 (2.54%) | 40,803 (2.71%) | 42,181 (2.37%) | 37,428 (2.51%) |
| MG | 530,306 (10.52%) | 148,016 (9.84%) | 185,997 (10.45%) | 158,227 (10.62%) |
| MS | 62,740 (1.24%) | 18,306 (1.22%) | 24,277 (1.36%) | 19,565 (1.31%) |
| MT | 66,805 (1.33%) | 22,216 (1.48%) | 27,412 (1.54%) | 20,333 (1.37%) |
| PA | 144,252 (2.86%) | 48,209 (3.20%) | 48,801 (2.74%) | 42,169 (2.83%) |
| PB | 104,069 (2.06%) | 30,030 (2.00%) | 33,582 (1.89%) | 31,175 (2.09%) |
| PE | 245,005 (4.86%) | 73,757 (4.90%) | 77,982 (4.38%) | 69,115 (4.64%) |
| PI | 74,763 (1.48%) | 22,592 (1.50%) | 25,190 (1.42%) | 23,229 (1.56%) |
| PR | 283,861 (5.63%) | 80,312 (5.34%) | 110,283 (6.20%) | 87,275 (5.86%) |
| RJ | 540,157 (10.72%) | 167,603 (11.14%) | 184,820 (10.38%) | 146,404 (9.83%) |
| RN | 82,136 (1.63%) | 23,874 (1.59%) | 25,950 (1.46%) | 23,387 (1.57%) |
| RO | 30.772 (0,61%) | 9,734 (0.65%) | 13,535 (0.76%) | 9,725 (0.65%) |
| RR | 8.593 (0,17%) | 3,154 (0.21%) | 3,840 (0.22%) | 2,819 (0.19%) |
| RS | 341.989 (6,78%) | 90,984 (6.05%) | 115,693 (6.50%) | 101,983 (6.85%) |
| SC | 157.913 (3,13%) | 45,104 (3.00%) | 58,512 (3.29%) | 49,806 (3.34%) |
| SE | 49.871 (0,99%) | 15,042 (1.00%) | 16,009 (0.90%) | 14,027 (0.94%) |
| SP | 1.153.841 (22,89%) | 341,123 (22.68%) | 422,948 (23.77%) | 344,666 (23.14%) |
| TO | 29.106 (0,58%) | 8,798 (0.58%) | 10,996 (0.62%) | 8,884 (0.60%) |

DM – Diabetes Mellitus; CVD – Cardiovascular Disease; HTN – Hypertention; AC - Acre; AL - Alagoas; AM - Amazonas; AP - Amapá; BA - Bahia; CE - Ceará; DF - Distrito Federal; ES - Espírito Santo; GO - Goiás; MA - Maranhão; MG - Minas Gerais; MS - Mato Grosso do Sul; MT - Mato Grosso; PA - Pará; PB - Paraíba; PE - Pernambuco; PI - Piauí; PR - Paraná; RJ - Rio de Janeiro; RN - Rio Grande do Norte; RO - Rondônia; RR - Roraima; RS - Rio Grande do Sul; SC - Santa Catarina; SE - Sergipe; SP - São Paulo; TO - Tocantins**.**
